# Blood Cell Counts and Inflammatory Indexes in Idiopathic Pulmonary Fibrosis

**DOI:** 10.7759/cureus.78319

**Published:** 2025-01-31

**Authors:** Damla Ay, Şeyma Başlılar, Gokce Kulah, Bengu Kaan Saylan, Gozde Kalbaran Kismet, Oguzhan Okutan

**Affiliations:** 1 Pulmonology, Kırsehir State Hospital, Kırsehir, TUR; 2 Pulmonology, Sultan 2. Abdülhamid Han Training and Research Hospital, Istanbul, TUR; 3 Pulmonology, University of Health Sciences, Istanbul, TUR; 4 Pulmonology, University of Health Sciences Sureyyapasa Chest Diseases and Thoracic Surgery Training and Research Hospital, Istanbul, TUR; 5 Pulmonology, Uskudar State Hospital, Istanbul, TUR; 6 Pulmonology, Maltepe University Hospital, Istanbul, TUR

**Keywords:** blood cells, idiopathic pulmonary fibrosis, inflammation indexes, mortality, prognosis.

## Abstract

Introduction

Inflammatory cells play a role in several idiopathic pulmonary fibrosis (IPF) pathogenesis steps. We aimed to evaluate the predictive value of peripheral blood cell (PBC) counts and inflammation indexes in the prognosis and mortality of IPF.

Materials and methods

A total of 155 patients with IPF followed between 1 January 2016 and 1 January 2023 were evaluated retrospectively. The baseline values and annual changes for pulmonary function tests and the PBC counts, ratios, and inflammation indexes (leukocyte, neutrophil, platelet, monocyte, lymphocyte, red cell distribution width (RDW), neutrophil-to-lymphocyte ratio (NLR), derived neutrophil-to-lymphocyte ratio (dNLR), platelet-to-lymphocyte ratio (PLR), monocyte-to-lymphocyte ratio (MLR), Systemic Immune Inflammation (SII) index, Systemic Inflammation Response Index (SIRI), the Aggregate Index of Systemic Inflammation (AISI)) were recorded. The relation between PBC, ratios, and inflammatory indexes with functional parameters (forced vital capacity (FVC), diffusing capacity of the lung for carbon monoxide (DLCO), 6-minute walking test (6MWT), Gender, Age, and Physiology (GAP) index, GAP stage) and mortality were examined.

Results

It was found that baseline RDW and neutrophil count were negatively correlated with survival time. The prognosis was worse in patients who had an RDW>13.6% and a neutrophil count>5.26×10^9^/L (p* *= 0.0005 and p = 0.037, respectively). Significant correlations were observed between baseline peripheral blood cell counts, ratios, and index values (leukocyte, monocyte, neutrophil, platelet, monocyte, lymphocyte, NLR, PLR, MLR, SII, SIRI, AISI) and functional parameters (FVC, DLCO, 6MWT, GAP index, GAP stage). However, there was no significant correlation between the yearly changes.

Conclusions

Increased neutrophils and RDW may be related to the poor prognosis in IPF. Peripheral blood cell counts and inflammatory indices may provide useful information in identifying patients with worse functional status.

## Introduction

Idiopathic pulmonary fibrosis (IPF) is a chronic fibrosing interstitial lung disease that usually has a progressive course and unknown cause [1, 2]. The incidence increases with age and is most frequent in the 6th and 7th decades. The course of the disease is quite variable, and while the disease remains stable for years in some patients, a rapid deterioration is observed in others [3]. It is important to identify patients with poor prognosis early to refer them for lung transplantation. For this purpose, scoring systems including clinical, functional, radiological data, and pulmonary function test parameters are widely used [4-6]. However, in some patients, due to advanced age and poor functional capacity, reliable data may not be obtained from functional tests such as the 6-minute walking test (6MWT), diffusion capacity of the lung for carbon monoxide (DLCO), and forced vital capacity (FVC).

In recent years, peripheral blood cell counts, which may reflect the inflammatory status, have attracted intense attention as simple and inexpensive prognostic biomarkers [7-9]. Repetitive microinjuries explain the pathophysiology of IPF in the alveolar epithelium, which results in chronic inflammation and abnormal tissue repair, which leads to progressive fibrosis. Inflammatory cells are involved in several steps in this pathogenetic pathway [10]. Chronic systemic inflammation causes an increase in the number of neutrophils, monocytes, and platelets but a decrease in the number of lymphocytes [11-13]. It has been suggested that the cell count ratios and combined blood cell count indexes of inflammation may be superior to cell counts alone in assessing the degree of inflammation [7].

In this study, we aimed to assess whether peripheral blood cell counts (PBC) and inflammatory indices such as SII (Systemic Immune Inflammatory Index), SIRI (Systemic Inflammatory Response Index), and AISI (Aggregate Index of Systemic Inflammation) provide independent prognostic value in IPF and how they compare with conventional clinical markers such as the Gender-Age-Physiology (GAP) Index, GAP stage, 6MWT, FVC, and DLCO. 

## Materials and methods

One hundred and fifty-five patients with a diagnosis of IPF at the Sultan 2. Abdülhamid Han Training and Research Hospital Chest Diseases Clinic, Istanbul between 1 January 2016 and 1 January 2023 were retrospectively examined. The local ethics committee approved the study (dated 07.07.2023 with decision number 23/392). Demographic and clinical characteristics of the patients (comorbidities, smoking status, medications, and long-term oxygen treatment (LTOT), etc.) were obtained from medical files. Baseline and yearly follow-up values for FVC (%), DLCO (%), change in partial oxygen saturation (ΔSpO2) after 6-minute walking test (6MWT) and 6-minute walking distance (6MWD), peripheral blood cell counts (leukocyte, neutrophil, monocyte, lymphocyte, and platelet), RDW (red blood cell distribution width), GAP index, and GAP stage were recorded. Also, systemic inflammation indices were calculated for all subjects.

All of the parameters were compared in patients with and without mortality. The relationship between peripheral blood cell counts, ratios, and inflammation indices with mortality and functional parameters (FVC, DLCO, 6MWT results, GAP index, and GAP stage) was investigated. Also, the relation between annual change in functional and PBC parameters was studied.

The inclusion criteria were: IPF diagnosed according to the American Thoracic Society/European Respiratory Society (ATS/ERS) 2011, 2018, and/or 2022 IPF diagnostic guidelines for adult patients.

The exclusion criteria were: those with pneumonia with confirmed pathogen microorganisms or pulmonary thromboembolism, those with an active malignancy, those with severe renal and hepatic insufficiency, and those undergoing immunosuppressive therapy in the last 3 months, excluding corticosteroid therapy.

Pulmonary function test measurements were performed without pre-bronchodilation at rest by experienced personnel using WinspiroPRO computer-based spirometry (MiniSpir, Rome, Italy) following ATS/ERS guidelines. At least three measurements were performed and the FVC value was recorded by selecting the best result. Since pulmonary function test parameters may vary according to age, height, and weight, expected values were expressed as % values.

The carbon monoxide diffusion test (Viasy single breath method) was performed following the ATS/ERS guidelines. The DLCO value was corrected by the device according to gender, age, height, weight, and current hemoglobin values and expressed as % values.

The 6MWT was performed in a 30-meter corridor by experienced personnel following the ATS/ERS guidelines. Each patient's baseline SpO2 value was measured. The patient was asked to walk a distance of 30 meters in the corridor within a given time duration. The distance walked and SpO2 value were measured after the test.

The GAP index (Gender-Age-Predicted FVC and DLCO values) and GAP stages were calculated to estimate the patients' mortality risk at diagnosis. Being male is 1 point, being 61-65 years of age is 1 point, being over 65 years of age is 2 points, FVC (%) between 50-75 is 1 point and below 50 is 2 points, DLCO (%) between 36-55 is 1 point and 35 or below is 2 points, and the patient not being able to perform is determined as 3 points. If the total score is 0-3, Stage I is determined; 4-5 is Stage II; 6-8 is Stage III. Mortality is correlated with the GAP stage. 

Peripheral blood cell (PBC) counts of all patients were recorded at 6-month intervals, and inflammation indices (Neutrophil-to-lymphocyte ratio (NLR), derived neutrophil-to-lymphocyte ratio (dNLR), platelet-to-lymphocyte ratio (PLR), monocyte-to-lymphocyte ratio (MLR), SII, SIRI, AISI) were calculated (Table 1).

**Table 1:** Calculation of Inflammation Indexes

Index	Calculation Method
Neutrophil-to-lymphocyte ratio (NLR)	neutrophil count/lymphocyte count
Derived neutrophil-to-lymphocyte ratio (dNLR)	(White blood cell count (WBC) - lymphocyte count)/lymphocyte count
Platelet-to-lymphocyte ratio (PLR)	platelet count/lymphocyte count
Monocyte-to-lymphocyte ratio (MLR)	monocyte count/lymphocyte count
Systemic immune inflammation index (SII)	platelet count × neutrophil count/lymphocyte count
Systemic inflammation response index (SIRI)	monocyte count × neutrophil count/lymphocyte count
The Aggregate Index of Systemic Inflammation (AISI)	neutrophil count × platelet count × monocyte count/lymphocyte count

Our study was planned to evaluate the patients receiving nintedanib and pirfenidone separately and to examine the correlations of FVC, DLCO, 6MWT, and PBC count values and the change in index values to evaluate the response to treatment; however, since the patient cohort would be very small and meaningful results would not be obtained, such grouping could not be made.

Statistical analysis

The sample size was calculated as 84 using G*Power (Heinrich-Heine University, Dusseldorf, Germany) analysis. Descriptive statistics were presented as mean and standard deviation or median and interquartile range and frequency and percentage for continuous and categorical data, respectively. For normally distributed data, t-test and ANOVA were used to compare the two groups. For categorical data, the Chi-square test was used to compare two or more groups. For comparison of non-normally distributed data, the Wilcoxon test and Mann-Whitney U test were used. The correlation analysis was made by the Spearman correlation method. Descriptive statistics, statistical tests, and advanced visualizations were performed using the R statistical programming language (R Foundation for Statistical Computing, Vienna, Austria). R statistical computing software is free software with a programming language that is frequently used in scientific analysis. The analyses in the study were performed using the R Studio software (R Foundation for Statistical Computing, Vienna, Austria) with the necessary coding (with R script language). A value of p<0.05 was considered significant.

## Results

One hundred and seventeen male (75.5%) and 38 female patients (24.5%) with a mean age (SD) of 71 (8) years were evaluated. 8.4% were active smokers, 62.6 % were ex-smokers and the mean smoking amount (SD) was 23.5 (22.6) package years. 29% (45 cases) were using long-term oxygen treatment (LTOT). 33.5% (52 cases) suffered from exacerbations, and 43.9 % (68 patients) died who had a mean survival time (SD) of 21.5 (14.7) months. The mean DLCO (%) and FVC (%) (SD) were 53.4 (22) and (SD) 76.8 (18), respectively. The distribution of patients according to GAP stage was as follows: stage I: 38.7% (60 cases), stage II: 50.3% (78 cases), and stage III: 11% (17 cases). The mean GAP index (SD) was 3.9 (1.3). The mean 6MWD (SD) was 373.7 (115.3) m. And ΔSpO2 (SD) (%) was 5.8 (5.4). The mean SD or median (interrange) values for baseline peripheral blood cell counts (10^9^/L), ratios, and indexes were as follows: leukocyte 8.62 (7.11-10.1), monocyte 0.57 (0.43-0.7), neutrophil 5.26 (4.18-6.4), lymphocyte 2.21 (1.69-2.8), platelet 242.2 ± 67.7, RDW (%) 13.6 (13-14.4), NLR 2.2 (1.64-2.99), dNLR 2.64 (2.01-3.47), PLR 101.15 (80.56-133.1), MLR (SD) 0.4 (0.8), SII 506.81 (385.24-763.59), SIRI 1.26 (0.82-1.83) and AISI 286.58 (190.93-466.74) (Table 2).

**Table 2:** Demographic, clinical and laboratory characteristics of idiopathic pulmonary fibrosis patients 6MWT: Six-Minute Walk Test, SpO2: peripheral oxygen saturation; AF: atrial fibrillation, AISI: Aggregate Index of Systemic Inflammation, BMI: Body mass index, CAD; Coronary artery disease, DLCO: diffusing capacity of the lung for carbon monoxide, DM: Type 2 Diabetes Mellitus, dNLR: Derived neutrophil-to-lymphocyte ratio, FVC: Forced vital capacity, HF: Heart failure, HT: hypertension, IPF: Idiopathic pulmonary fibrosis, LTOT: Long-term oxygen treatment, MLR: Monocyte-to-lymphocyte ratio, NLR: Neutrophil-to-lymphocyte ratio, PHT: Pulmonary hypertension, PLR: Platelet-to-lymphocyte ratio, RDW: Red blood cell distribution width, SII: Systemic immune inflammation index, SIRI: Systemic inflammation response index, GAP:  Gender-Age-Physiology Index

Characteristic	Value (N=155)
Age (years)	71 ± 8
Gender (Female/Male)	38 (25% /117 (75%)
BMI (kg/m²)	26.2 ± 2.8
Smoking history (active smoker/former smoker/never smoked)	13 (9%)/97 (6%)/45 (29%)
Cigarette (package-years)	23.5 ± 22.6
Exitus/Alive	68 (44%)/87 (56%)
Post-diagnosis survival of dead patients (months)	41 ± 33
Exacerbation history (yes/no)	52 (34%)/103 (66%)
LTOT (yes/no)	45 (29%)/110 (71%)
DLCO%	53.4 ± 22
FVC%	76.8 ± 18
GAP Index	3.9 ± 1.3
GAP Stage	I: 60 (39%) / II: 78 (50%) / III: 17 (11%)
6MWT Walking Distance (meters)	373.5 ± 115.3
6MWT SPO2 Change	5.8 ± 5.4
Comorbidities	
CAD	73 (47%)
AF	19 (12.3%)
HT	108 (69.7%)
DM	66 (42%)
Chronic respiratory disease	8 (5.25)
HF	21 (13.5%)
Malignancy	6 (3.9%)
PHT	64 (41.3%)
Medication use	
Only nintedanib	20 (13%)
Only pirfenidone	82 (53%)
Medication changes	30 (19%)
Never used	23 (15%)
Laboratory values	
Leukocyte 10⁹/L	8.62 (7.1-10.1)
RDW%	13.6 (13-14.4)
Monocyte 10⁹/L	0.57 (0.43-0.7)
Neutrophil 10⁹/L	5.26 (4.18-6.4)
Lymphocyte 10⁹/L	2.21 (1.69-2.8)
Platelet 10⁹/L	242.2 ± 67.7
Indexes	
NLR	2.2 (1.64-2.99)
dNLR	2.64 (2.01-3.47)
PLR	101.15 (80.56-133.1)
MLR	0.4 ± 0.8
SII	506.81 (385.24-763.59)
SIRI	1.26 (0.82-1.83)
AISI	286.58 (190.93-466.74)

**Figure 1:**
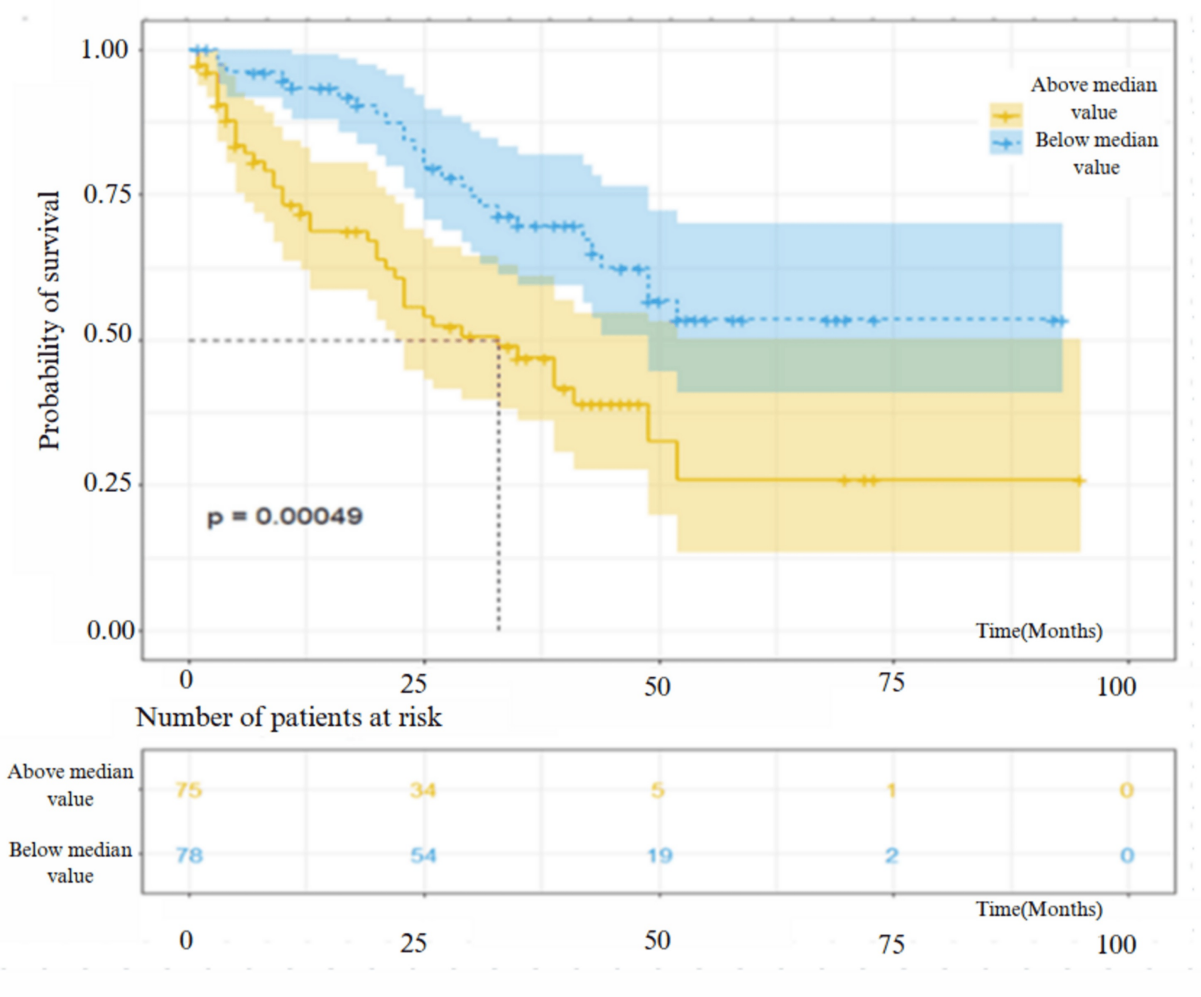
Survival analysis of patients with IPF included in the study according to values above and below the median value of RDW. IPF: idiopathic pulmonary fibrosis; RDW: red cell distribution width

**Figure 2:**
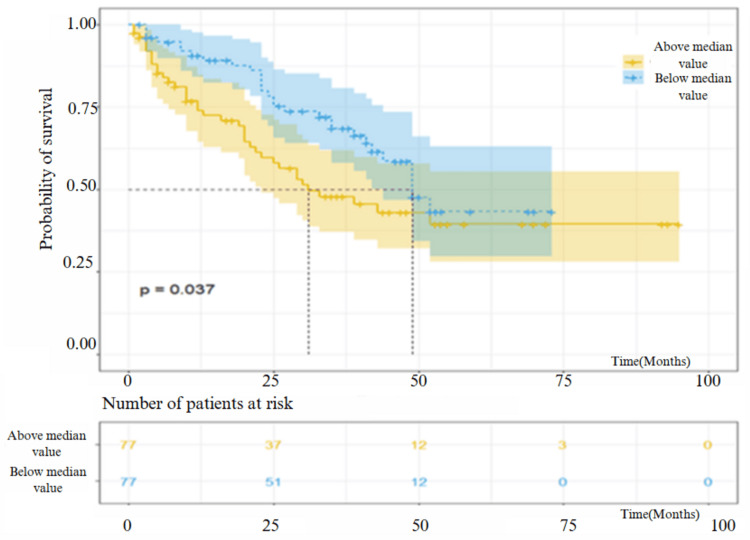
Survival analysis of the patients included in the study according to values below and above the neutrophil median value.

The survival analysis showed a significant decrease in survival rate in patients who had a median RDW value above 13.6 and neutrophil count above 5.26 (p = 0.0005 and p = 0.037, respectively), while the other PBC counts, ratios, and inflammatory indexes were not correlated with the survival (p > 0.05) (Table 3). The survival analysis graph according to the median values of RDW and neutrophils is shown in Figure 1 and Figure 2.

**Table 3:** Survival analysis according to median values for peripheral blood cell count values and inflammation indices (LogRank Test) WBC: white blood cell, RDW: red cell distribution width, NLR: neutrophil-to-lymphocyte ratio, dNLR: derived neutrophil-to-lymphocyte ratio, PLR: platelet-to-lymphocyte ratio, MLR: monocyte-to-lymphocyte ratio, SII: Systemic Immune Inflammation Index, SIRI: Systemic Inflammation Response Index, AISI: Aggregate Index of Systemic Inflammation.

Values	Median	Chi-Square	p-value
Leukocyte 10^9^/L	8.62	1.907	0.167
RDW %	13.6	12.160	0.0005
Monocyte 10^9^/L	0.57	1.183	0.277
Neutrophil 10^9^/L	5.26	4.336	0.037
Lymphocyte 10^9^/L	2.21	1.521	0.217
Platelet 10^9^/L	241	0.841	0.359
NLR	2.2	2.473	0.116
dNLR	2.64	3.52	0.06
PLR	101.15	0.000	0.995
MLR	0.24	2.741	0.097
SII	506.81	0.245	0.62
SIRI	1.26	2.217	0.136
AISI	286.58	0.252	0.615

The correlations between the baseline peripheral blood cell counts and baseline functional parameters are shown in Table 4. There were significant negative correlations between DLCO and leukocyte (p = 0.008, r = -0.213), RDW (p = 0.005, r = -0.226), monocyte (p = 0.035, r = -0.171), neutrophil (p = 0.038, r = -0.168), and platelet counts (p = 0.036, r = -0.171). There were significant negative correlations between FVC and leukocyte (p = 0.035, r = -0.171), RDW (p = 0.007, r = -0.221), and neutrophil counts (p = 0.043, r = -0.165). 6MWD was negatively correlated with RDW (p = 0.041, r = -0.167) and neutrophil count (p = 0.036, r = -0.171), while GAP index and GAP stage were positively correlated with leukocytes, RDW, and neutrophils (p = 0.039, r = 0.166 and p = 0.006, r = 0.223 and p = 0.012, r = 0.203, respectively for GAP index, and p = 0.019, r = 0.188 and p = 0.029, r = 0.176 and p = 0.011, r = 0.205, respectively for GAP stage.

**Table 4:** Correlation between baseline peripheral blood cell count values and DLCO (%), FVC (%), 6MWD, GAP Index, and GAP Stage values 6MWD: 6-minute walk distance, DLCO: Diffusing capacity for carbon monoxide, FVC: Forced vital capacity, p: Probability value, r: Correlation value, RDW: Red cell distribution width, GAP: Gender-Age-Physiology index

Values		DLCO (%)	FVC (%)	6MWD	GAP Index	GAP Stage
Leukocyte 10^9^/L	r	-0.213	-0.171	-0.158	0.166	0.188
	p	0.008	0.035	0.053	0.039	0.019
RDW %	r	-0.226	-0.221	-0.167	0.223	0.176
	p	0.005	0.007	0.041	0.006	0.029
Monocyte 10^9^/L	r	-0.171	-0.154	-0.062	0.098	0.078
	p	0.035	0.059	0.452	0.225	0.337
Neutrophil 10^9^/L	r	-0.168	-0.165	-0.171	0.203	0.205
	p	0.038	0.043	0.036	0.012	0.011
Lymphocyte 10^9^/L	r	-0.012	0.088	0.114	-0.127	-0.074
	p	0.884	0.283	0.163	0.116	0.363
Platelet 10^9^/L	r	-0.171	-0.129	-0.122	0.084	0.096
	p	0.036	0.113	0.136	0.301	0.238

The correlations between the baseline peripheral blood cell count ratio index values and baseline functional parameters are shown in Table 5. DLCO was negatively correlated with SIRI (p = 0.031, r = -0.175) and AISI (p = 0.03, r = -0.176), FVC was negatively correlated with NLR (p = 0.03, r = -0.177), PLR (p = 0.004, r = -0.232), dNLR (p = 0.004, r = -0.232), SII (p = 0.015, r = -0.198), SIRI (p = 0.001, r = -0.261), and AISI (p = 0.004, r = -0.233), and 6MWD was negatively correlated with NLR (p = 0.027, r = -0.18), PLR (p = 0.016, r = - 0.196), SII (p = 0.015, r = -0.198), and SIRI (p = 0.028, r = -0.18). There were positive correlations between GAP index and GAP stage with NLR (p = 0.005), PLR (p = 0.0008), dNLR (p = 0.026), SII (p = 0.005), SIRI (p = 0.002), and AISI (p = 0.007) for GAP index, and NLR (p = 0.013, r = 0.201), PLR (p = 0.003, r = 0.235), SII (p = 0.008, r = 0.214), SIRI (p = 0.01, r = 0.208), and AISI (p = 0.023, r = 0.184) for GAP stage.

**Table 5:** Correlation between index values and DLCO (%), FVC (%), 6MWD, GAP Index, and GAP Stage 6MWD: 6-minute walk distance, AISI: Aggregate index of systemic inflammation, DLCO: Diffusing capacity for carbon monoxide, dNLR: Derived neutrophil to lymphocyte ratio, FVC: Forced vital capacity, MLR: Monocyte-to-lymphocyte ratio, NLR: Neutrophil-to-lymphocyte ratio, p: Probability value, PLR: Platelet-to-lymphocyte ratio, r: Correlation value, SII: Systemic Immune Inflammation Index, SIRI: Systemic Inflammation Response Index; GAP:  Gender-Age-Physiology index

Values		DLCO (%)	FVC (%)	6MWD	GAP Index	GAP Stage
NLR	r	-0.111	-0.177	-0.180	0.226	0.201
	p	0.175	0.030	0.027	0.005	0.013
PLR	r	-0.144	-0.232	-0.196	0.268	0.235
	p	0.076	0.004	0.016	0.0008	0.003
MLR	r	-0.062	-0.147	0.160	0.143	0.109
	p	0.447	0.071	0.051	0.077	0.180
dNLR	r	-0.090	-0.232	-0.132	0.180	0.135
	p	0.269	0.004	0.108	0.026	0.094
SII	r	-0.145	-0.198	-0.198	0.227	0.214
	p	0.074	0.015	0.015	0.005	0.008
SIRI	r	-0.175	-0.261	-0.180	0.245	0.208
	p	0.031	0.001	0.028	0.002	0.010
AISI	r	-0.176	-0.233	-0.159	0.216	0.184
	p	0.030	0.004	0.052	0.007	0.23

In patients receiving antifibrotic treatment, correlations between post-treatment yearly changes in FVC, DLCO, and 6MWD and inflammation indices were examined in the first year of treatment. A weak negative correlation was observed between the ΔFVC (%) and ΔMLR (p = 0.043, r = -0.224), Δ6MWD and ΔWBC (p = 0.035, r = -0.251), and Δneutrophil counts (p = 0.005, r = -0,328).

The number of patients followed decreased gradually after the first year, and no significant relations can be achieved for the changes in the second to fifth years.

## Discussion

The study detected significant correlations between the functional parameters, PBC counts, and index values. Leukocyte count was negatively correlated with DLCO and FVC and positively correlated with the GAP stage. Neutrophil count was negatively correlated with FVC, DLCO, and 6MWD and positively correlated with the GAP stage. A significant negative correlation was observed between monocyte count and DLCO. Survival was lower in patients with a baseline neutrophil count above 5.26 and RDW value above 13.6. 

Various studies show that PBC counts reflect inflammation in IPF [8, 14, 15]. In a multicenter study, it was reported that leukocyte count showed a negative correlation with FVC and DLCO, similar to our findings, and the risk of death increased 1.85 times in the group with increased leukocyte count [16]. These findings suggest that the increased leukocyte count reflects disease-related inflammation and may be associated with disease severity, functional loss, and mortality.

Neutrophils are important inflammatory cells in the pathogenesis of IPF [17]. The results of our study showed that the baseline neutrophil count was negatively correlated with functional parameters and associated with the disease's severity. Furthermore, the mortality rate was higher in those with increased neutrophils. This suggests that neutrophil count may indicate the clinical course, outcome, and mortality of the disease. There are conflicting results in the literature about the relationship between the percentage of neutrophils in bronchoalveolar lavage (BAL) fluid and mortality. In one study, increased neutrophil percentage was shown to be an important determinant of early mortality [18]. In contrast, Veeraraghavan et al. reported no relationship between BAL fluid neutrophil percentage and survival [19].

Monocytes are the circulating precursors of macrophages. It has been suggested that in IPF, monocytes migrate from the bone marrow to the damaged lung and transform into fibrotic macrophages, and the increase in monocytes is associated with the progression of fibrosis [14]. It was reported that the mortality rate was higher in IPF patients with a monocyte count > 0.95 [8, 20]. In our study, the median monocyte count was 0.57 and not related to mortality, possibly due to the lower cut-off value compared to the previous studies.

Previous studies showed that RDW is associated with mortality not only in IPF patients but also in the general population [21]. Also, various studies have shown that there is a relationship between RDW and right ventricular dysfunction and pulmonary hypertension (PHT), which are important risk factors for mortality in IPF patients [22, 23]. It was suggested that RDW may provide useful information in predicting mortality. The increase in RDW is explained by chronic/intermittent hypoxemia, which may indicate underlying pulmonary hypertension. The decrease in partial pressure of oxygen (PaO2) stimulates erythropoiesis, which results in a change in RDW [24]. Besides, tissue hypoxia triggers an inflammatory response and oxidative stress that disrupts the erythrocyte cycle, leading to anisocytosis [25]. Although IPF patients with chronic and profound hypoxemia can be easily identified, intermittent hypoxemia may not be recognized during routine examinations. Increased RDW with normal SpO2 at rest may point to patients with intermittent hypoxemia.

Nathan et al. made a study of 319 IPF patients; it was found that the RDW value was negatively correlated with survival, and the survival was lower in patients who had a higher monthly increase in RDW [22]. In another multicenter study, it was reported that patients with RDW values ≥14.1 had lower FVC and DLCO values. RDW was significantly related to disease progression but not related to mortality [8]. In our study, the median RDW value was 13.6, and an RDW ≥13.6 was an independent factor in predicting survival. A significant correlation was detected between baseline RDW and functional parameters, which may reflect the severity of the disease, but no significant correlation was found between annual RDW changes, which may be explained by the significant decrease in the number of patients in years. These results suggest that RDW may be related to the disease severity in IPF.

In the inflammation in IPF, there is an increase in neutrophils, monocytes, and platelets and a decrease in lymphocytes [12, 13]. It was proposed that inflammatory cell count ratios would reflect inflammation better than cell counts alone [7]. In our study, NLR and PLR were negatively correlated with FVC and 6MWD and positively correlated with the GAP stage at the time of diagnosis. No correlation was observed between MLR and functional parameters. dNLR was negatively correlated with FVC and positively correlated with GAP index. In previous studies, it has been shown that there was a negative correlation between NLR in BAL fluid and FVC [26]. It has been reported that the cut-off values related to worse prognosis were 6.4 and 250.5 for NLR and PLR, respectively, and the mortality rate and decline in functional parameters were higher in patients with higher NLR and PLR at diagnosis. Besides, the clinical outcomes (mortality, hospitalization for respiratory reasons, decrease in lung functions, and exercise capacity) were worse in patients with greater annual increases in NLR and PLR at follow-up. In another study, it was reported that the NLR on the last visit before death was significantly higher than the NLR at the time of diagnosis (SD) 21.52 (13.48) vs. (SD) 15.17 (16.96), P=014). There was a negative correlation between NLR, PLR, and PaO2/fraction of inspired oxygen (FiO2), but no relationship was found with MLR. These results suggest that NLR may be a more valuable biomarker in predicting mortality than PLR and MLR [9].

It is suggested that combined indices may reflect chronic inflammation better since several inflammatory cell types were considered. However, conflicting results have been reported in the literature, and combined indices have not been proven yet to be more useful than other indices [7, 27]. It was reported that the mean SII values were similar between interstitial lung disease secondary to connective tissue diseases (CTD-ILD) and IPF groups and between survivors and non-survivors with IPF [12]. In our study, we also failed to show any difference in the median SII values between the patients who died and survived. In a study conducted by Zinellu et al. with 73 patients with IPF and 62 healthy controls, NLR, dNLR, LMR, SIRI, and AISI were associated with the presence of IPF. Additionally, there were significant correlations between inflammation indices and functional parameters. So, the authors suggested that the inflammation indices may be related to IPF severity [7]. In another study, FEV in 1 second (FEV1) and FVC (%) and 6MWD were lower in patients with AISI≥434 [27]. In both studies, there was a significant relationship between index values and mortality.

In our study, negative correlations were observed between SII, SIRI, AISI, and functional parameters, and a positive correlation was observed between SII, SIRI, AISI, and the GAP index. These results suggest that combined inflammatory indices may reflect the disease severity. However, unlike previous studies, no significant relationship was observed between these indices and mortality. This may be explained by the retrospective design of the study; conditions such as steroid use, unreported infections, or exacerbations that might affect the PBC counts may be ignored, and these may in turn change the combined index values.

Due to the short life expectancy in IPF, data on long-term follow-up are inadequate. There are a few studies investigating changes in PBC counts and ratios during long-term follow-up. Nathan et al. evaluated the correlation between the change in functional parameters and PBC counts and inflammation indices in IPF patients receiving antifibrotic treatment. The patients were divided into quartiles based on 12th-month median NLR and PLR changes, and worse clinical outcomes were observed in the group with the greatest change [28].

To our knowledge, our study is the first in the literature to examine the correlation between changes in functional parameters and index values. Similar studies have been previously conducted with KL-6, MMP-7, SP-D, CA19-9, CA-125, and periostin [29, 30]. Monitoring these inflammatory markers is both expensive and not available in routine clinical practice. In our study, the changes in leukocyte and neutrophil counts were related to the change in 6MWD, and the change in MLR was related to the change in FVC (%) in the first year. In the third year, only monocyte change was associated with FVC change. The mean follow-up period was 30 months; 44% of the patients died during follow-up, and the survival time of the deceased patients after diagnosis was 20 months. So, the number of patients followed up for more than 2 years was quite low. For this reason, evaluation for long-term follow-up is inadequate. Multicenter prospective studies with more patients are necessary to enlighten this subject.

The limitations of our study are that it is a single-center study with a retrospective study design, and as the number of patients gradually decreased during long-term follow-up due to the high mortality rate, long-term analysis of the parameters was inadequate. On the other hand, complete blood count parameters can be easily affected by inflammatory conditions such as infection, steroid use, and pulmonary embolism and can vary from day to day. Although we recorded the laboratory parameters at least 3 months following an acute exacerbation or corticosteroid use, there might be unreported conditions.

## Conclusions

We concluded that increased baseline neutrophils and RDW may be related to the poor prognosis in IPF. Peripheral blood cell counts and inflammatory indices at diagnosis may provide useful information in identifying patients with worse functional status, but multicenter, prospective clinical studies are necessary to determine their prognostic value as biomarkers in long-term follow-up.
